# Achieving research impact in medical research through collaboration across organizational boundaries: Insights from a mixed methods study in the Netherlands

**DOI:** 10.1186/s12961-024-01157-z

**Published:** 2024-06-25

**Authors:** Jacqueline C. F. van Oijen, Annemieke van Dongen-Leunis, Jeroen Postma, Thed van Leeuwen, Roland Bal

**Affiliations:** 1 Erasmus School of Health Policy & Management, Erasmus University Rotterdam, P.O. Box 1738, 3000 DR Rotterdam, The Netherlands; 2 Centre for Science and Technology Studies, Leiden University, Leiden, The Netherlands

**Keywords:** Collaboration, Research impact, Bibliometric analysis, Organizational boundary work

## Abstract

**Background:**

In the Netherlands, university medical centres (UMCs) bear primary responsibility for conducting medical research and delivering highly specialized care. The TopCare program was a policy experiment lasting 4 years in which three non-academic hospitals received funding from the Dutch Ministry of Health to also conduct medical research and deliver highly specialized care in specific domains. This study investigates research collaboration outcomes for all Dutch UMCs and non-academic hospitals in general and, more specifically, for the domains in the non-academic hospitals participating in the TopCare program. Additionally, it explores the organizational boundary work employed by these hospitals to foster productive research collaborations.

**Methods:**

A mixed method research design was employed combining quantitative bibliometric analysis of publications and citations across all Dutch UMCs and non-academic hospitals and the TopCare domains with geographical distances, document analysis and ethnographic interviews with actors in the TopCare program.

**Results:**

Quantitative analysis shows that, over the period of study, international collaboration increased among all hospitals while national collaboration and single institution research declined slightly. Collaborative efforts correlated with higher impact scores, and international collaboration scored higher than national collaboration. A total of 60% of all non-academic hospitals’ publications were produced in collaboration with UMCs, whereas almost 30% of the UMCs’ publications were the result of such collaboration. Non-academic hospitals showed a higher rate of collaboration with the UMC that was nearest geographically, whereas TopCare hospitals prioritized expertise over geographical proximity within their specialized domains. Boundary work mechanisms adopted by TopCare hospitals included aligning research activities with organizational mindset (identity), bolstering research infrastructure (competence) and finding and mobilizing strategic partnerships with academic partners (power). These efforts aimed to establish credibility and attractiveness as collaboration partners.

**Conclusions:**

Research collaboration between non-academic hospitals and UMCs, particularly where this also involves international collaboration, pays off in terms of publications and impact. The TopCare hospitals used the program’s resources to perform boundary work aimed at becoming an attractive and credible collaboration partner for academia. Local factors such as research history, strategic domain focus, in-house expertise, patient flows, infrastructure and network relationships influenced collaboration dynamics within TopCare hospitals and between them and UMCs.

## Introduction

Research collaboration has taken flight worldwide in recent decades [1], as reflected by the growing number of authors listed on research papers [2, 3]. Collaborative research has become the norm for many, if not most, scientific disciplines [4–8]. Several studies have found a positive relationship between collaboration and output [9–13]. Publications resulting from research collaborations tend to be cited more frequently [14–18] and to be of higher research quality [5, 14, 19, 20]. In particular, international collaboration can lead to more citations [17, 21–24], although there are major differences internationally and between fields [25]. Moreover, international collaboration is often set as an eligibility requirement for European research grants, which have become necessary as national-level resources dwindle. Funding consortia also encourage and require boundary crossings, such as research collaborations between academia and societal partners. Collaboration within public research organizations and universities further plays a crucial role in the international dissemination of knowledge [26].

In the medical domain, initiatives have been rolled out in numerous countries to encourage long-term collaboration and the exchange of knowledge and research findings. Each initiative takes a strategic approach to assembling the processes needed to support these exchanges across the boundaries of stakeholder groups. In the Netherlands, medical research has traditionally been concentrated in public academia, especially the university medical centres (UMCs). Increasingly, however, research activities are being undertaken in non-academic teaching hospitals (hereafter, non-academic hospitals), driven by their changing patterns of patient influx. In 2013, a Dutch study based on citation analysis showed that collaboration between UMCs and non-academic hospitals leads to high-quality research [27]. There was further encouragement for medical research in Dutch non-academic hospitals in 2014, when a 4-year policy experiment, the TopCare program, was launched, with three such hospitals receiving additional funding from the Ministry of Health to also provide highly specialized care and undertake medical research. Funding for this combination of care and research is available for UMCs under the budgetary “academic component” of the Dutch healthcare system. Such additional funds are not available for non-academic hospitals, nor can they allocate their regular budgets to research. In the past, these hospitals managed to conduct research and provide specialized care through their own financial and time investments, or by securing occasional external research funding. The TopCare policy experiment was thus meant to find new ways of organizing and funding highly specialized care and medical research in non-academic hospitals.

Despite the increasing emphasis on research collaboration, we still know little about its impact and how it can be achieved. This study integrates two sides of research collaboration in Dutch hospitals and combines elements of quantitative and qualitative research for a broad (output and impact) and deep (boundary work to achieve collaboration) understanding of the phenomenon. We define research collaboration as collaboration between two or more organizations (at least one being a UMC or non-academic hospital) that has resulted in a co-authored (joint) scientific publication [28]. The research questions are: How high is the level of collaboration in the Dutch medical research field, what is the impact of collaboration, and how are productive research collaborations achieved?

To answer these questions, we performed mixed methods research in UMCs and non-academic hospitals. To examine the impact of various collaboration models – namely, single institution, national and international – across all eight Dutch UMCs and 28 non-academic hospitals between 2009 and 2018/2019, we conducted a bibliometric analysis of publications and citations. We additionally carried out a similar analysis for the TopCare non-academic hospitals between 2010 and 2016 to examine the effects of collaboration in the two domains funded through the program at each hospital. The latter timeframe was chosen to match the duration of the program, 2014–2018. We further conducted an in-depth qualitative analysis of the organizational boundary work done by two non-academic hospitals participating in the TopCare program to initiate and enhance productive research collaborations around specialized research and care within and between hospitals on a national level. Historically, such endeavours have been predominantly reserved for UMCs. The program was therefore a unique opportunity to examine such boundary work.

## Background and theory

### The landscape of medical research in the Netherlands

#### Collaboration in medical research

The Netherlands has a three-tiered hospital system: general hospitals (including non-academic hospitals), specialized hospitals focusing on a specific medical field or patient population, and UMCs. Nowadays, there are 7 UMCs, 17 specialized hospitals and 58 general hospitals, of which 26 are non-academic [29].

UMCs receive special funding (the budgetary “academic component”) for research and oversee medical training programs in their region. Non-academic hospitals do not receive structural government funding for medical research and have less chance of obtaining other funding because they are not formally acknowledged as knowledge-producing organizations. Research has less priority in most of these hospitals than in UMCs. On the introduction of government policies regarding competition in healthcare and the development of quality guidelines emphasizing high-volume treatments, some non-academic hospitals began focusing on specific disease areas, in a bid to distinguish themselves from other hospitals and to perform research in and hence develop more knowledge about these priority areas. This led to a greater concentration of highly specialized care [30]. Non-academic hospitals have also become important partners in medical research for UMCs due to their large patient volumes.

#### The TopCare program

To further stimulate research in non-academic hospitals, the Ministry of Health awarded three such hospitals €28.8 million in funding over a 4-year period (2014–2018) to support medical research and specialized care for which they do not normally receive funding [31]. It should be noted that, in non-academic hospitals, the concept of highly specialized research and care applies not to the entire hospital but rather to specific departments or disease areas. This is why the TopCare non-academic hospitals have been evaluated on the basis of specific domains. The funding recipients were two non-academic hospitals and one specialized hospital. In this article, we focus on UMCs and general non-academic hospitals and therefore excluded the specialized hospital from our analysis. Hospital #1 is the largest non-academic hospital in the Netherlands (1100 beds), even larger than some UMCs. Its fields of excellence (known as “domains”) are lung and heart care. Hospital #2 is a large non-academic hospital (950 beds) that focuses on emergency care and neurology. According to the two hospitals, these four highly specialized care and research-intensive domains are comparable to high-complexity care and research in UMCs [31].

The TopCare program ran through ZonMw, the Netherlands Organization for Health Research and Development, the main funding body for health research in the Netherlands. ZonMw established a committee to assess the research proposals and complex care initiatives of the participating hospitals and to set several criteria for funding eligibility. One requirement was that participating hospitals had to collaborate with universities or UMCs on research projects and were not allowed to conduct basic research in the context of the program, as this was seen as the special province of UMCs.

#### Boundary work

In the qualitative part of this study, we analyse the boundary work done by actors to influence organizational boundaries as well as the practices undertaken to initiate or enhance collaboration between TopCare non-academic hospitals and academia (universities and UMCs). We refer to boundary work when actors create, shape or disrupt organizational boundaries [32–35]. In particular, boundary work involves opening a boundary for collaboration and creating linkages with external partners [36]. In this article, we use three organizational boundary concepts – “identity”, “competence” and “power” – out of four presented by Santos and Eisenhardt. These concepts are concerned with fostering collaboration, whereas the fourth is concerned with “efficiency” and is less relevant here. Identity involves creating a reputation for research to become an attractive partner while preserving identity. Competence involves creating opportunities for research, for example, in manpower and infrastructure. Finally, power involves creating a negotiating position vis-à-vis relevant others [35].

### Methods

The data for this study consist of different types of analysis: (1) quantitative bibliometric data on the publications and citations of all eight Dutch UMCs and 28 non-academic hospitals, and (2) quantitative bibliometric data on the publications and citations in the four domains of two TopCare non-academic hospitals, qualitative (policy) document analysis and in-depth ethnographic interviews with various actors in the Dutch TopCare program. The quantitative data collected from Dutch UMCs and non-academic hospitals were utilized to contextualize data gathered within the TopCare program. We discuss and explain the data collection and methodology in detail in the two sections below.

1. Quantitative approach: bibliometric analysis of all 8 Dutch UMCs and 28 non-academic hospitals

#### Data collection

We performed a bibliometric analysis of the publications of 28 non-academic hospitals and 8 UMCs^1^ in the Netherlands between 2009 and 2018. Data for the study were derived from the Center for Science and Technology Studies’ (CWTS) in-house version of the Web of Science (WoS) database. The year 2009 was chosen because the address affiliations in publications are more accurately defined from this year onward. To examine trends over time, we divided the period 2009–2018/2019 into two blocks of 4 years and an additional year for citation impact measurement (2009–2012/2013 and 2014–2017/2018; see explanation in Appendix 1).

#### Methodology

The bibliometric analysis includes several bibliometric indicators that describe both the output and impact of the relevant research (Table 5 in Appendix 1). One of the indicators, the mean normalized citation score (MNCS), reveals the average impact of a hospital’s publications compared with the average score of all other publications in that area of research. If the MNCS is higher than 1, then on average, the output of that hospital’s domain is cited more often than an “average” publication in that research area.

To map the ways hospitals cooperate, we follow two lines of analysis. The first is centred around a typology of scientific activities and differentiates between (i) a single institution (SI;  all publications with only one address) and (ii) international collaboration (IC; collaboration with at least one international partner). All other publications are grouped as (iii) national collaboration (NC; collaboration with Dutch organizations only).

The second line is centred around geographical distance and size of collaboration. The geographical distances between each non-academic hospital and each of the eight UMCs were measured in Google Maps. The size of collaboration was measured by counting the joint publications of each non-academic hospital and the eight UMCs. Subsequently, we assessed whether the non-academic hospitals also had the most joint publications with the nearest UMC.

[2] Quantitative and qualitative approach to the two TopCare hospitals and their four domains, the “TopCare program” case study

#### Data collection

##### Quantitative approach

The quantitative approach to the TopCare program relies on a bibliometric analysis of publications within each hospital’s two domains: lung and heart care in TopCare non-academic hospital #1, and trauma and neurology in TopCare non-academic hospital #2. Our bibliometric analysis focused on publications within the four selected TopCare domains between 2010 and 2016, following the same methodology described in the previous section under ‘Data collection’. Each domain provided an overview of its publications. The number of publications produced by the two domains at each TopCare hospital is combined in the results. Although this timeframe differs from the broader analysis of all UMCs and non-academic hospitals, comparing these two periods offers insights into the “representative position” of the two domains of each non-academic hospital participating in the TopCare program, in terms of publications and citations.

##### Qualitative approach

We took a qualitative approach to analysing the collaborative activities in the two TopCare non-academic hospitals, where each domain has its own leadership arrangements, regional demographic priorities and history of research collaboration [cf. 37]. This part of the study consisted of interviews and document analysis.

##### Ethnographic interviews

Over the course of the 4-year program, J.P. and/or R.B. conducted and recorded 90 semi-structured interviews that were then transcribed. For this study, we used repeated in-depth ethnographic interviews with the main actors in the Dutch TopCare program, which took place between 2014 and 2018. We conducted a total of 27 interviews; 20 of the interviews were with a single person, 5 with two persons, and 2 with three persons. The interviews were held with 20 different respondents; 12 respondents were interviewed multiple times. Table 1 presents the different respondents in non-academic hospitals #1 and #2.

**Table 1:** Number of interviews with TopCare program actors for this study

Non-academic hospital #1 Lung and heart care	*N*	Number of times interviewed	Non-academic hospital #2 Emergency care and neurology	*N*	Number of times interviewed
Board of directors	1	2×	Board of directors	1	2×
Project and program leaders TopCare program	2	1: 2× 1: 3x	Project and program leaders TopCare program	3	1: 1× 2: 3x
Healthcare managers	2	2: 1×			
Researchers (2 medical specialists and 1 professor)	3	1: 1× 1: 2x 1: 3×	Researchers (2 post-docs, 3 medical specialists and 3 professors)	8	4: 1× 4: 2×
	8			12	

##### Document analysis

Desk research was performed for documents related to the TopCare program (Table 6 – details of document analysis in Appendix 1).

#### Methodology

##### Quantitative approach

The bibliometric analysis of the four domains in the two TopCare non-academic hospitals follows the same methodology as described in Abramo et al. [1].

We tested the assumption that joint publications are most frequent between a non-academic hospital and its nearest UMC. If the geographical distance between TopCare non-academic hospitals and their collaborative academic partners is described as “nearby”, then they both work within the same region.

##### Qualitative approach

The ethnographic interviews were audio-recorded and transcribed in full with the respondents’ permission. These transcripts were subject to close reading and coding by two authors, J.P. and J.O., to identify key themes derived from the theory [35] (Table 7 in the Appendix). These were then discussed and debated with the wider research team with the goal of developing a critical interpretation of the boundary work done to initiate or enhance research collaboration [cf. 37]. The processed interview data were submitted to the respondents for member check. All respondents gave permission to use the data for this study, including the specific quotes. In the Netherlands, this research requires no ethical approval.

Triangulating the results of the document analysis and the interviews enables us to identify different overarching themes within each boundary concept (identity, competence and power). These themes were utilized as a framework for structuring individual paragraphs, which we explain in greater detail in Table 4 in the Results.

## Results

1. Bibliometric analysis of all Dutch UMCs and non-academic hospitals

This section reports the results of the quantitative bibliometric analysis of the output, trends and impact of collaboration between all UMCs and non-academic hospitals from 2009 to 2018/2019. It provides a broad picture of the output – in terms of research publications – of both existing and ongoing collaborations between all UMCs and non-academic hospitals within the specified timeframe. It furthermore describes the analysis results concerning the relationship between collaboration and the geographical distance between two collaborating hospitals.

### Output: distribution of the types of collaboration for UMCs and non-academic hospitals from 2009 to 2018/2019

**Fig. 1:**
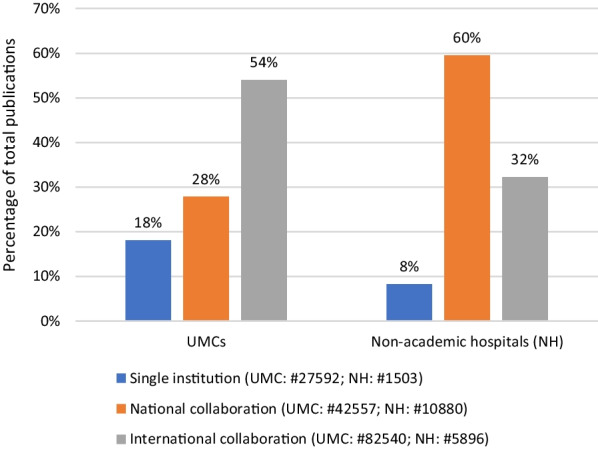
Types of collaboration for UMCs and non-academic hospitals from 2009 to 2018/2019. ^#^Total number of publications. Percentage of total (100%) accounted for by single institution, national collaboration and international collaboration

The first step in understanding the degree of collaboration between hospitals is to measure the research output by number of publications. The total number of publications between 2009 and 2018 is shown in Table 8 ( Appendix 1) and Fig. 1.

The majority of these publications (89%) are affiliated with UMCs. UMCs, in particular, tend to have a relatively higher proportion of single-institution publications and are more engaged in international collaboration. This pattern may be indicative of UMCs’ enhanced access to research grants and EU subsidies, as well as their active involvement in international consortia.

Collaboration between UMCs and non-academic hospitals appears to be more prevalent and impactful for non-academic hospitals than for UMCs: 70% of all publications originating from a non-academic hospital were the result of joint efforts between a UMC and a non-academic hospital, whereas only 8% of all UMC publications were produced in collaboration with a non-academic hospital (Table 8 in Appendix 1).

### Trend analysis of collaboration in relative number of publications

**Fig. 2:**
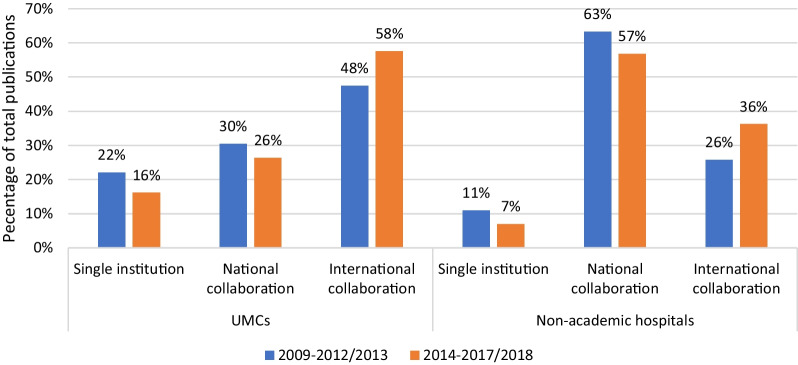
Type of research collaboration for UMCs and non-academic hospitals over time. Percentage of total (100%) accounted for by single institution, national collaboration and international collaboration in each period

Table 9 Appendix 1) and Fig. 2 show the relative number of publications of all 8 UMCs and all 28 non-academic hospitals in the two periods: 2009–2012/2013 and 2014–2017/2018. For both UMCs and non-academic hospitals, international collaboration accounted for a relatively larger share of publications in recent years.

### Analysis of relationship between distance and collaboration

As the non-academic hospitals often collaborate with UMCs, it is interesting to analyse these collaborations geographically (distance). The assumption is that geographical proximity matters, with the most-frequent joint publications being between a non-academic hospital and the nearest UMC.

**Fig. 3:**
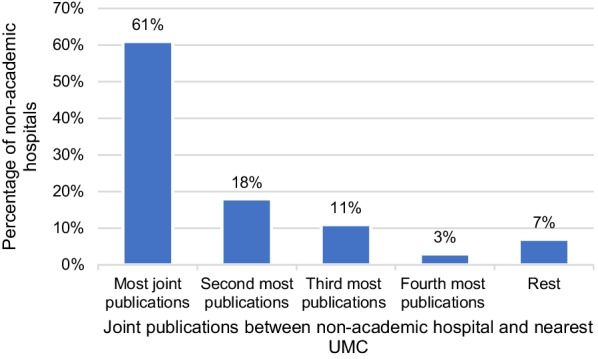
Collaboration with nearest UMC from 2009 to 2018

Figure 3 shows that 61% (17 out of 28) of the non-academic hospitals collaborate most frequently with the nearest UMCs. Geographical proximity is thus an important but not the only determining factor in collaboration.

### Impact of collaboration on bibliometric output of UMCs and non-academic hospitals

The mean normalized citation scores (MNCS) shown in Table 2 cover all 8 UMCs and 28 non-academic hospitals.

**Table 2:** MNCS

	2009–2018/2019				2009–2012/2013				2014–2017/2018			
	UMCs	Number	Non-academic hospitals	Number	UMCs	Number	Non-academic hospitals	Number	UMCs	Number	Non-academic hospitals	**Number**
SI	1.27	27,592	0.90	1503	1.32	11,204	0.93	704	1.24	11,085	0.85	559
NC	1.20	42,557	1.22	10,880	1.28	15,468	1.35	4060	1.17	18,087	1.17	4556
IC	2.05	82,450	2.24	5896	2.08	24,133	2.16	1656	2.01	39,493	2.32	2908

If the MNCS is higher (or lower) than 1, then on average, the output of the domain is cited more often (or less often) than an “average” publication in the research area in which the domain is active. “Number” refers to the total number of publications

SI, single institution; NC, national collaboration; IC, international collaboration

The MNCS in Table 2 and the mean normalized journal scores (MNJS) in Table 10 (Appendix 1) show similar patterns. The impact score for both UMCs and non-academic hospitals is greatest for international collaboration. Non-academic hospitals’ single-institution publications score lower than the global average, which was defined as 1.

In sum, quantitative analysis exposes two trends. The first is growth in international collaboration for all UMCs and non-academic hospitals over time, also revealing that collaboration leads to higher MNCS impact scores. Second, geographical proximity between UMCs and non-academic hospitals is an important but not the only determining factor in collaboration. This is the context in which the TopCare program operated in 2014–2018.

2. “TopCare program” case study

This section presents the results of our analysis of the collaboration networks of the two TopCare non-academic hospitals, consisting of: (1) quantitative bibliometric analysis of the output and impact of these networks between 2010 and 2016, along with the geographical distance to their academic partners, and (2) qualitative ethnographic interviews to identify the boundary work conducted by these hospitals.

1. Bibliometric analysis of the two TopCare non-academic hospitals’ international and national collaboration networks across four domains

**Fig. 4:**
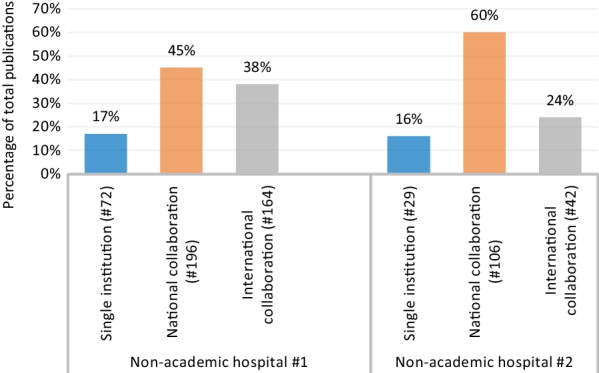
Types of collaboration involving TopCare hospitals #1 and #2 between 2010 and 2016. #, total number of publications

The results of the bibliometric analysis indicate the representative positions of the two domains within each TopCare non-academic hospital. Between 2010 and 2016, these hospitals generated a higher number of single-institution publications compared with the average of all non-academic hospitals. Percentage-wise, their output resembled that of the UMCs, underscoring their leading positions in their respective domains. The percentage of publications based on national collaboration in the domains of TopCare hospital #2 is comparable to that of non-academic hospitals overall, while there is more international collaboration in the domains of TopCare hospital #1 than at non-academic hospitals overall (Fig. 4, Appendix 1 and Fig. 1). The impact of the research is above the global average, and the publications have a higher average impact when there is collaboration with international partners; this is true across all four domains (Table 11 in Appendix 1).

In terms of geographical distance, only the neurology domain of TopCare hospital #2 collaborates with an academic partner within the same region. All other domains collaborate with partners outside the region, a striking difference from the geographical results shown in Fig. 3.

2. Ethnographic analysis

This section reviews the results of our ethnographic analysis of the two TopCare hospitals from 2014 to 2018. To analyse the boundary work these hospitals performed to initiate and/or enhance productive research collaborations, we use the framework suggested by Santos and Eisenhardt (2005) for examining organizational boundary work through the concepts of identity, competence and power. Table 3 provides a description of each boundary and how these concepts are defined in our case study on the basis of the overarching themes in the document analysis and the interviews.

**Table 3:** Description of each boundary to case study specifications

	Description of each boundary (based on [38, 39]) case study specifications	Case study specifications
Boundary of identity	Maintain coherence between the hospital’s dominant mindset of “who we are” and its organizational activities	(a) Enhancing the hospitals’ value proposition
Boundary of competence	Maximize the value of a hospital’s resource portfolio by matching resources with opportunities of its collaborative partners	(b) Finding alignments within hospitals and research networks
Boundary of power	Maximize strategic control over crucial relationships, and increase the hospital’s power in a particular domain	(a) Enhancing the relationship with or finding and mobilizing strategic academic partners (b) Aligning with the board of directors and administrators of the TopCare hospitals

### Identity: enhancing hospitals’ value proposition

In the TopCare program, the non-academic hospitals used their unique history and expertise to create a joint research focus in a domain and to enhance their positions and influence their collaboration with UMCs and universities.

A manager in hospital #1’s lung domain explained the work being done from a historical perspective, emphasizing not only the innovative history of the hospital but also its central position in patient care:

The first-ever lung lavage, lung transplant and angioplasty were performed in this hospital. Nationally, this hospital has always, and we’re talking about 50–60 years ago now, been at the forefront, and has always invested in this line of research and care. So that is truly institutionally built, there is just that history and you can’t just copy that. And we have the numbers: for interstitial lung diseases, we have 2000 patients in our practice and receive 600 new patients per year. (interview with manager at hospital #1 in 2018).

To explain why patient care and research into rare interstitial lung diseases is centred in hospital #1 as a strategic domain focus, a leading international pulmonary physician – a “boundary spanner” (see below) – pointed to the importance of building team expertise and creating facilities:

I lead that care program for interstitial lung diseases and preside over the related research. I’ve often been asked: you’re a professor, so why don’t you go to a UMC, couldn’t you do much more there? But the care was developed here [in this hospital]. The expertise needed to recognize interstitial lung diseases depends not only on me but also on the radiologist and pathologist; together we have a team that can do this. We have created facilities that no other hospital has for these diseases. If I leave to do the same work in a UMC, I’d have to start over and I’d be going back 30 years. (interview with pulmonary physician at hospital #1 in 2014).

The doctors working in this hospital’s lung and heart domains finance the working hours they put into research themselves. “This fits in with the spirit of a top clinical hospital and the entrepreneurial character of our hospital.” (interview with project leader at hospital #1 in 2018).

Hospital #2, the result of a merger in 2016, struggled to find its strategic focus. A surgical oncologist at this hospital clarified one of the disadvantages of the merger: “People are [still] busy dealing with the money and positions, and the gaze is turned inward, the primary processes. So clinical research is very low on the agenda.” She continued by saying that a small project team acting on behalf of the hospital’s board of directors (BoD) was seeking the best-fit profile for the program, which had raised some opposition in departments excluded from the chosen strategic focus. As a consequence, the hospital had begun to showcase its highly specialized care in the field of neurosurgical treatments. It had a long history and was the first to use a Gamma Knife device for treating brain tumours. The experts in this domain could thus act as authorities, and they became a national centre of expertise. Their strategic partner was a nearby UMC, and they treated relevant patients from other hospitals in their region.

To generate impact, research priorities in a domain are aligned with the focus of the hospital. A member of the BoD of hospital #2 stressed the urgency of “specializing or focusing on a particular area of care” and emphasized that the TopCare budget was being utilized to create a joint focus within a domain. The resulting collective identity mobilized internal affairs and was recognized as valuable by third parties. An important reason for joining the TopCare program for both hospitals was to be able to position themselves strategically as attractive and credible research partners:

The focus is on the domains of neurology and trauma because we think as a non-academic hospital we have something extra to offer: the very close relationship between patient care and research, because we have a larger number of patients of this type here than the universities. (interview with care manager at hospital #2 in 2013).

In short, the boundary of identity requires a closer alignment between these hospitals’ research activities and their strategic objectives and organizational mindset, and demands that they also showcase their staff’s expertise. The TopCare program offered opportunities to transform and consolidate their identity by enhancing their value proposition, that is, their unique history, strategic domain focus, expertise and number of patients.

### Competence: Enhancing research infrastructures

All domains in the TopCare program chose to utilize the TopCare funding to invest in their research infrastructure, and to build research networks to share and learn. A research infrastructure consists of all the organizational, human, material and technological facilities needed for specialist care and research [31].

The TopCare data show that funding is essential for generating research impact. A manager at hospital #1 described its current financial circumstances:

A lot of research and much of the care is currently not funded, it is actually paid for mostly by the hospital... We have had massive budgetary adjustments the past two or three years. ...It is increasingly difficult to finance these kinds of activities within your own operation. (interview with manager at hospital #1 in 2018).

The TopCare funding was used to enhance the material infrastructure in hospital #1’s heart domain:

A number of things in healthcare are really terribly expensive, and there is simply no financing at all for them. …Cardiac devices, for example. We are constantly trying things out, but there’s no compensation for it. (interview with project leader at hospital #1 in 2018).

Hospital #1 had a long-standing and firm relationship with a UMC in the lung domain, giving it a solid material infrastructure. For example, there were spaces where researchers, especially PhD students, could meet, collaborate and share knowledge [31]. Another essential part of the material infrastructure for the lung domain was the biobank, as highlighted by a leading international pulmonary physician:

Our board of directors made funds available through the innovation fund to start up a biobank, but developing it and keeping it afloat has now been made possible thanks to the TopCare funding. It’s a gift from heaven! It will allow for further expansion and we can now seek international cooperation. (interview with pulmonary physician at hospital #1 in 2014).

Notably, the program allowed both non-academic hospitals to digitize their infrastructure, for example, with clinical registration and data management systems. According to an orthopaedic surgeon at hospital #2, “Logistics have been created, which can very easily be applied to other areas. By purchasing a data system, everyone can record data in a similar way.”

Besides investing in data infrastructure, the human dimension was another crucial factor in the research infrastructure. Instead of working on research “at night”, it became embedded in physicians’ working hours. All domains indicated the importance of having researchers, statisticians and data management expertise available to ensure and enhance the quality of research, and both hospitals invested in research staffing.

After losing many research-minded traumatologists to academia, hospital #2 decided to invest in dedicated researchers to form an intermediate layer of full-time senior researchers linked to clinicians within the two domains.

I personally think this is the most important layer in a hospital, with both a doctor and a senior researcher supervising students and PhD candidates. Clinicians ask practical questions and researchers ask a lot of theoretical questions. Both perspectives are needed to change practices. I have also learned that it takes a few years before the two can understand each other’s language. (interview with neurosurgeon at hospital #2 in 2018).

### Competence: Finding alignments within hospitals and research networks

The program offered the hospitals opportunities to structure internal forms of collaboration and build a knowledge base within a domain. For example, hospital #1 organized educational sessions with all PhD students in the heart domain.

Having more researchers working in our hospital has given the whole research culture a boost, as well as the fact that they are producing more publications and dissertations. (interview with cardiologist at hospital #1 in 2018).

Hospital #2 also encouraged cross-domain learning by organizing meetings between the neurology and trauma domains.

You know, you may not be able to do much together content-wise, but you can learn a lot from each other in terms of the obstacles you face (interview with project manager at hospital #2 in 2016).

At the beginning there was resistance to participating in the program.

It was doom and gloom; without more support, groups refused to join. That kind of discussion. So the financial details have been important in terms of willingness to participate. (interview with surgical oncologist at hospital #2 in 2018).

Another obstacle was local approval for multicentre studies, which led to considerable delay (interview with psychologist at hospital #2 in 2018). Overall, the TopCare program created a flywheel effect for other domains that proved essential for internal collaborations (interview with surgical oncologist at hospital #2 in 2018).

In hospital #1, collaboration between the heart and lung domains grew closer.

Divisions between the different disciplines are much less pronounced in our hospital than in UMCs. So it’s much easier to work together. We’d already collaborated closely on lung diseases, and this has improved during the program. (interview with cardiologist at hospital #1 in 2016)

At the network level, the TopCare data show that most researchers participated in national networks. For example, the neurology domain in hospital #2 had established a network of 16 non-academic hospitals. Limited funding prevented researchers at non-academic hospitals from attending many international seminars, and they had more trouble building their international networks. One exception concerned the researchers in the lung domain of hospital #1, who expanded their international network by organizing an international seminar during the TopCare program and by contributing to other national and international seminars.

Each TopCare domain provided highly specialized care and wanted to become a centre of expertise. However, a hospital can only provide highly specialized care if research is conducted to determine the best treatment strategies. The data show how the two are interwoven.

For example, a PhD student has sought to collaborate with a UMC on a specific aorta subject in which we have greater expertise and more volume in terms of patients than UMCs. Based on this link with this UMC, a different policy was drawn up and also implemented immediately in all kinds of other UMCs. (interview with cardiologist at hospital #1 in 2018).

Often, a leading scientist who is the driving force behind a domain in a hospital is a “boundary spanner”, a person in a unique position to bridge organizational boundaries and foster research collaboration by “enabling exchange between production and use of knowledge” [40, p. 1176], [41]. For example, the leading pulmonary physician in hospital #1 is a boundary spanner who has done a huge amount of work to enhance collaboration. With interstitial lung disease care being concentrated here, this professor can offer fellowships and stimulate virtual knowledge-sharing by video conferencing for “second-opinion” consultations. The TopCare funding was used to finance this. The network is successful at a non-academic level.

These consultations are with colleagues in other hospitals and they avoid patients having to be referred. (interview with project leader at hospital #1 in 2018).

Our network now [in 2018] consists of more than 14 hospitals, which we call every week to discuss patients with an interstitial lung disease. …UMCs participate indirectly in this network. For example, the north has a specific centre for this disease in a non-academic hospital and a nearby UMC refers patients to this centre, who are then discussed in our network. (interview with pulmonary physician at hospital #1 in 2018).

This physician also noted that the network was still growing; other colleagues from non-academic hospitals wanted to join it.

Yesterday, colleagues from XX and XX were here. And they all said, “I’ve never learned so much about interstitial lung diseases.” We’re imparting enormous amounts of expertise. (interview with pulmonary physician at hospital #1 in 2018).

In sum, focusing on the boundary of competence, the TopCare hospitals created and mobilized resources to invest in their research infrastructure. In every domain, this infrastructure was used to strengthen the relationship between research, care and education, and to build and enhance internal and external research networks to share and learn.

### Power: Enhancing the relationship with or finding and mobilizing strategic academic partners

For TopCare non-academic hospitals, the boundary of power is concerned with creating the right sphere of influence, meaning BoDs and administrators attempt to find and mobilize new strategic partners and build mutual relationships with various stakeholders at different levels.

A project leader at hospital #2 emphasized that the additional resources of the TopCare program created an opportunity for the non-academic hospitals “to show our collaborative partners that we’re a valuable partner.” For once, the tables were turned:

We’ve always had a good relationship with one UMC; they always used the data from our surgeries. But it’s nice that we can finally ask them whether they want to join us. That makes it a little more equal, and we can be a clinical partner. (interview with neurosurgeon at hospital #2 in 2018).

One of the requirements in each domain when applying to ZonMw for funding was alignment with academia in a research and innovation network. Collaboration often appeared more difficult at the administrative level when the academic partners worked in the same field of expertise, and tended to be more successful when the partners focused on different fields, where their interests did not conflict. According to a board member at hospital #2 who played a crucial role in a partnership agreement, a conscious decision was taken beforehand to seek partners beyond the medical domain as well.

There may be conflict with other groups within the walls of a UMC and I don’t see that as promising. You have to work together and we aren’t in a real position to do so. (interview with board member at hospital #2 in 2018).

Just before the end of the program, it was announced that this hospital had concluded a partnership agreement with a university to broaden their joint research program alongside neurology and trauma. An important prerequisite was that both organizations invest 1 million euros in the partnership. The board member revealed that the relationship with this university had in fact existed for some time:

So we went and talked to the university and they became interested. Then the top level was reorganized and replaced and we had to start from scratch again. That took a lot of time. Our goals were to awaken the enthusiasm of the board and at least three deans, otherwise it would be a very isolated matter. And we succeeded. Last week we had a matchmaking meeting at the university and there were about 50 pitches showing how we could be of value to each other. (interview with board member at hospital #2 in 2018)

Looking back, he defined the conditions for a successful collaboration with academia:

In terms of substance, the two sides have to be going in the same direction and complement each other, for example, in expertise, techniques, and/or facilities. And what is really important is that people know each other and are willing to meet each other…and there must be appreciation. (interview with board member at hospital #2 in 2018).

The trauma domain in hospital #2 wanted to become a trauma research centre in its region, and after investing in its research infrastructure, it found a new strategic academic partner:

We have also found new partners, for example, the Social Health Care Department of a UMC [name]. And that really has become a strong partnership; the intent was there for years, but we had no money. (interview with epidemiologist at hospital #2 in 2018).

The neurology domain at this hospital worked to form a network with a university of technology and a university social science department.

Officially, our hospital can’t serve as a co-applicant for funding and that is frustrating. However, I am pleased to show that we are contributing to innovation. (interview with neurosurgeon at hospital #2 in 2018).

A board member at this hospital reflected on the qualities needed for research and concluded: “The neuro group has more of those intrinsic qualities than the trauma group. …I think the trauma group is actually at a crossroads and will think twice about whether they can attract capacity to develop the research side or fall back to a very basic level.”

In hospital #1, administrators rejected a proposal to collaborate with the nearest UMC submitted by medical specialists in the heart domain. Past conflicts and unsuccessful ventures still influenced the present, even though the individuals involved had already left.

A further factor was raised by a manager at hospital #1, who reflected on the importance of obtaining a professorship in the heart domain:

If we can, even on the basis of any kind of appointment, obtain a professorship from the heart centre, then yes, that helps! …I think it just helps throughout the whole operation, politically speaking, as extra confirmation, extra legitimization for that status. (interview with manager at hospital #1 in 2016).

Eventually, hospital #1 managed to find alignment with a UMC in another region during the program and a medical specialist from the hospital became a professor by special appointment.

This UMC showed the greatest determination, actually, while we could have chosen to collaborate with the nearest UMC [but we didn’t]. And there was actually also a real click between both the administrators and the specialists. (interview with manager at hospital #1 in 2018).

Additionally, the TopCare data show that, while there may be close alignment with the nearest UMC, collaboration is not limited to this and proximity can sometimes even be detrimental (for example, in some cases hospitals compete for patients). As research and care in the TopCare hospitals’ domains became more specialized, they required the specific expertise of UMCs in other regions.

One critical dependency in the collaboration between a university or UMC and a non-academic hospital is the distribution of dissertation premiums, valued at about €100,000 per successful PhD track. Currently, after completion of a dissertation, the premium goes entirely to the university or UMC, even when much of the candidate’s research and supervision takes place in a non-academic hospital [31]. This structural difference makes collaboration less financially valuable to non-academic hospitals. For example, the leading pulmonary physician in hospital #1 is a professor who is affiliated with both a UMC and non-academic hospital, a boundary spanner who works across organizational boundaries, is successful in research, and bears responsibility for a significant proportion of the research output in the lung domain and in the collaboration with other organizations. Moreover, he does most of the PhD supervision, and his students do their work in hospital #1. Despite all this work, the dissertation premium goes to the UMC. Although efforts have been made to change this, certain institutional structures are so strongly embedded that it is difficult to open the organizational boundary.

### Power: Aligning with the BoDs and administrators of the TopCare non-academic hospitals

During our research, we observed how the BoDs and administrators of the two TopCare hospitals discussed the progress of the program and worked together to learn from each other.

We can learn a lot from hospital #1 regarding the organization of our research, we think. That has been very inspiring. …On the other hand, the focus has been very centred on getting the domain and project requests funded at all. (interview with care manager at hospital #2 in 2013).

The BoDs opted for an approach aimed at building mutual trust and understanding. As a result, their alliance became more intensive during the program. By the time the program’s final report was released, both BoDs were leveraging their power to influence ZonMw’s next step: the follow-up to TopCare. They had a targeted plan for their lobbying. For example, after mutual coordination, the BoD of each hospital sent a letter to the Ministry of Health sketching their vision for the future.

In summary, for the TopCare hospitals, the boundary of power centred on finding alignment with strategic academic partners and the other BoDs and administrators in the TopCare program. Moreover, ties with strategic partners were important for extending the organization’s sphere of influence [33] in building and enhancing productive research collaborations. These hospitals recognized that they could not dismantle the existing structure of research funding, and they therefore committed themselves to trying to extend the TopCare program. Table 4 summarizes the opportunities and challenges within the three boundary concepts.

**Table 4:** Opportunities and challenges within the three boundary concepts

Section of analysis	Opportunities	Challenges
Boundary of identity	- Enhance value proposition by integrating unique history, strategic focus, expertise and patient volume	- Fund doctors’ research hours - Manage disruptions from hospital mergers
Boundary of competence	Strengthen research–care–education synergy: - invest in research infrastructure, enhance materials, expand human resources and digitalize - Build and enrich both internal/external research networks for knowledge-sharing and learning	- Allocate research infrastructure resources - Stakeholder resistance - Limited funding hampers international networking
Boundary of power	- Establish conducive environment for partnerships: ensure equitable financial contributions or acquire professorships - Achieve success with diverse partners: minimize conflicts and expand beyond medicine - Cultivate relationships with BoDs and administrators - Consolidate authority to influence ZonMw’s TopCare follow-up	- Insufficient funding for doctors’ research time - Co-applicant limitations for funding - Past conflicts influence partnerships - Complicating factor: dissertation rewards to universities/UMCs

## Discussion

In our study, we used a mixed methods research design to explore research collaborations by focusing on the research output and impact of UMCs and non-academic hospitals in the Netherlands and by zeroing in on the boundary work of two Dutch non-academic hospitals for achieving collaboration.

Our bibliometric analysis shows that collaboration matters, especially for non-academic hospitals. Access to research grants, EU funding and international collaborations is harder for non-academic hospitals, and they need to collaborate with UMCs to generate research impact, assessed by means of MNCS impact scores. Conversely, non-academic hospitals are important for UMCs because they have a larger volume of patients. When UMCs and non-academic hospitals collaborate, their impact scores are higher. Impact scores are, moreover, higher for international collaborative publications across all types of hospital and all periods. More in-depth research is needed into why collaboration increases impact.

Bibliometric analysis of the domains of the two TopCare non-academic hospitals underscores their leading role in these domains. Upon receiving TopCare funding, the hospitals had to engage in various forms of boundary work to meet the requirement mandated by ZonMw of establishing a research collaboration with academia. They used the additional program resources to invest [33] in opening a boundary for research collaboration with academic partners.

Identity work involves creating an image of the organizational unit that legitimizes its research and care status in line with the dominant mindset of the organization. In practice, the relevant unit needs to establish a distinctive history and domain focus that aligns with the organizational strategy of the hospital, in-house expertise and patient flow. This requires coordination work with the BoD. However, not all domains have been successful in creating such an identity. It proved much more difficult for the trauma domain, for example, because their research is not as highly specialized as and more fragmented than the other domains.

Competence work focuses on organizational (a well-functioning science support unit), technological (registration systems) and material (floor space or biobank) infrastructure, depending on individual requirements. Additionally, tremendous efforts go into the human dimension of infrastructure, as TopCare hospitals consider research staff and making time available for doctors to be important conditions for building structurally supportive research programs. In a previous study, we highlighted that collaboration between all non-academic hospitals within the Association of Top Clinical Teaching Hospitals (STZ) is essential for strengthening their research infrastructure [42], and can also be seen as a matter of efficiency [35]. Moreover, in each TopCare hospital, competence work served to bring domains together to facilitate shared learning. Knowledge-sharing across departments or communities is an example of opening boundaries to facilitate integration, convergence or enrichment of points of view [36, 43, 44].

Professors with double affiliations can act as boundary spanners. They play a significant role as experts in a domain by creating its distinctive character, and they surmount borders and break down barriers through their network relationships with other hospitals. Additionally, these persons are responsible for a significant share of the research output in their domain and conduct research with worldwide impact in collaboration with other organizations. Their boundary work must be recognized as essential because they bring usable knowledge to the table, create opportunities for improved relationships across disciplines, enhance communication between stakeholders and facilitate more productive research collaborations [cf. 45].

The TopCare hospitals do much less work in the power dimension because the domains in which they operate are adjacent to those of academia. Our study shows that more successful, productive research collaborations are created when the hospital’s academic partner works in a complementary but not identical field. Only in one case, the heart domain, did collaboration succeed in an identical field, but that was because the academic partner was located outside of the hospital’s region and was therefore not a competitor. According to Joo et al., a potential partner’s suitability is determined not only by complementarity, their unique contribution to research collaboration in terms of expertise, skills, knowledge, contexts or resources but also by compatibility and capacity. Partner compatibility involves alignment in vision, commitment, trust, culture, values, norms and working styles, which facilitate rapport-building and cross-institutional collaboration [46]. TopCare data indicate that research collaborations should be managed to ensure all partners can operate as equals [47]. Partner capacity refers to the ability to provide timely resources (for example, expertise, skills or knowledge) for projects, as well as leadership commitment, community engagement and institutional support for long-term, mission-driven goals, such as the joint research program in neurology and trauma at hospital #2 and a university.

These three qualitative criteria – partner compatibility, complementarity and capacity – are aspects of power dynamics that influence strategic decisions about recruiting research partners. Generally, power dynamics shape a hospital’s strategic choices regarding whether to collaborate, with whom to partner and the extent of the research collaboration [48]. Future research should examine these power dynamics in a more integrated manner to unlock the full potential of collaboration [46].

It was possible to unravel how non-academic hospitals participating in the TopCare program engaged in research collaborations with academia. As the program did not interfere with the existing care, research and financing structures within the UMCs, it allowed TopCare non-academic hospitals to also combine top clinical care and research. The boundary concepts allow us to observe a dual dynamic in the collaboration: the opening of boundaries while simultaneously maintaining certain limits. Opening boundaries refers to facilitating collaboration through activities related to identity and competence, while maintaining them involves the power balance. The temporary program did not disrupt the existing power balance associated with the budgetary “academic component” and the dissertation premiums that accrue to academia. Overall, then, the power dimension may well be the primary factor that made it impossible for the TopCare non-academic hospitals to attain their ultimate goal: secure a consistent form of funding for their research and top clinical care. Instead, the national authorities introduced a new, temporary funding program for non-academic hospitals, and preserved the status quo favouring academia.

A key finding is that, if a hospital is successful in establishing coherence between the different forms of boundary work, it can create productive research collaborations and generate research impact. The TopCare hospitals performed boundary work to strengthen their research infrastructure (competence) and their research status (identity) and create a favourable negotiating position opposite academia (power). For example, choosing the lung domain as the hospital’s strategic focus (identity) and establishing a database as a fundamental source of information for research by a boundary spanner (competence) generated sufficient power to make the hospital a key player in this field and a much-respected collaboration partner, nationally and internationally. However, some restrictions remained in place, such as the national lung research network consisting only of non-academic hospitals, with UMCs participating only indirectly.

Another key finding is that possessing a substantial budget is not in itself enough to ensure successful research collaboration. It is clear from this study that extensive boundary work is also needed to facilitate research collaboration. Given the absence of structural funding, the TopCare non-academic hospitals were under pressure to deliver results during the program, making research collaboration even more crucial for them than for the UMCs in this context. Additionally, because highly specialized care and research at the TopCare non-academic hospitals required unique expertise, they had a growing need for collaboration at the national level. Contrary to assumptions and the findings of our analysis of UMCs and non-academic hospitals overall, their collaborative partners were not predominantly located at the nearest UMC.

Does our study align with the literature and support the results of similar initiatives, such as the establishment of Collaborations for Leadership in Applied Health Research and Care (CLAHRC), a regional multi-agency research network of universities and local national health service (NHS) organizations focused on improving patient outcomes in England by conducting and utilizing applied health research [49]? And what does it contribute to previous research?

While differences exist between the National Health Service (NHS) and the healthcare system in the Netherlands, there are also noteworthy parallels that render a comparison possible. These include encouraging networks to boost research productivity, fostering collaboration within a competitive system and funding research that is relevant to public health priorities. Moreover, building upon the findings of CLAHRC regarding boundary work within a competitive system and developing and funding research that is relevant to patient needs and public health priorities, there are further parallels, such as creating strong local research infrastructures and local networks [49], and using influential and skilled boundary spanners [49, 50]. In addition, we found that research history, strategic domain focus, in-house expertise, patient flows, and network relationships pre-conditioned the TopCare hospitals’ collaboration with academia. Our results further show that, for non-academic hospitals seeking to create productive research collaborations, it is essential to work in complementary fields and to establish a coherence between identity, competence and power.

Our findings indicate that, after opening a boundary with academia, the focus of the TopCare hospitals was on searching for mutual engagement. These hospitals tried to clarify their added value by creating boundaries to distinguish themselves from UMCs, and attempted to extend the TopCare program without it overlapping with the budgetary “academic component”, so that it posed no threat to the UMCs. Boundary-crossing involves a two-way interaction of mutual engagement and commitment to change in practices [51]. It is likely that the program did not last long enough to instigate changes in practices, as it can take time to develop mutual understanding and foster trusting relationships [52].

Based on the CLAHRC results and our research findings, the trend towards regionalization in the Netherlands [53] and a new leading and coordinating role for UMCs in this research landscape [52, 54] can only be successful if boundary work is conducted, allowing research-minded non-academic hospitals to:

- Build a “collaborative identity” [50, 55, 56] over time with their academic partners (identity);
- Establish added value in their research infrastructures compared with that of their academic partners (competence);
- Create solid networks for learning and sharing knowledge [55, 57] with their academic partners (competence);
- Mobilize boundary spanners to bridge disciplinary and professional boundaries in research, teaching and practice [49, 50, 55, 58] and publish articles in collaboration with academic partners with high research impact (competence);
- Find the inspiration and confidence to increase their co-dependence to, for example, gain benefits from interacting with different partners in the field [35] (power); and
- Create long-term collaborations with academia across sectors over time, as well as within sectors; this requires iterative and continual engagement between clinicians, academics, managers, practitioners and patients (power) [49, 52].

It is conceivable that the evaluation of the follow-up study to the TopCare program, which will extend to 2025, could unravel these next steps.

Our results demonstrate that collaboration in research is important and should be encouraged. However, the current methods used to assess researchers underestimate this importance. Reward systems and metrics focus on the performance of individual researchers and may even discourage the development of medical research networks and collaboration [52, 59]. There is ongoing debate about and rising criticism of the dominance of scientific impact scores as a measure of the performance of health researchers and research organizations [60]. Other forms of impact, such as the societal impact of medical research, are becoming more important, and different metrics are being developed. Research collaboration among individuals and organizations should be incentivized and rewarded, and should also be embedded in performance assessment and the core competences of all actors involved [61]. New ways of rewarding research collaboration within organizations should therefore be explored.

### Limitations

This study is limited, both geographically and institutionally, to the Netherlands, and factors other than national and international research collaborations may explain the increase in research output and impact. For example, the research articles in our sample have not been analysed on substantive aspects such as methodology and funding. A bias may therefore have been introduced. Furthermore, the research output and impact of the TopCare non-academic hospitals that we measured was limited to the 4-year program period. A further limitation was the use of these hospitals’ research output as a measure of the influence of the TopCare program, as we were interested not only in the short-term effects (publications) but also in the long-term ones (on the work conducted to build research infrastructures). Moreover, the focus in the qualitative material concerning the TopCare program was on the two TopCare non-academic hospitals and, more specifically, on their national rather than their international collaborations.

## Conclusions

Research collaboration between non-academic hospitals and academia in the Netherlands pays off in terms of publications and impact. For the publication of scientific articles, collaboration between UMCs and non-academic hospitals appears to be more prevalent and impactful for non-academic hospitals than for UMCs. When UMCs and non-academic hospitals collaborate, their impact scores tend to be higher. More research is needed into why collaboration leads to more impact.

Non-academic hospitals showed a higher rate of collaboration with the nearest UMC, whereas collaborative partners of TopCare hospitals were not predominantly located at the nearest UMC. TopCare hospitals prioritized expertise over geographical proximity as a predicator of their collaborative efforts, particularly as research and care in their domains became more specialized.

Drawing on the additional resources of the TopCare program, participating non-academic hospitals invested significantly in boundary work to open boundaries for research collaboration with academic partners and, simultaneously, to create boundaries that distinguished them from UMCs. Identity work was performed to ensure that their history and domain focuses were coherent with the dominant mindset of their organization, while competence work was done to enhance their research infrastructure. The human dimension of the infrastructure received considerable attention: more research staff, time made available for doctors and recognition that boundary spanners facilitate research collaborations.

Power work to find and mobilize strategic academic partners was mostly focused on complementary fields, as non-academic hospitals work in domains adjacent to those of academia. The TopCare hospitals tended to avoid power conflicts, resulting in a preservation of the status quo favouring academia.

The local research history, strategic domain focus, in-house expertise, patient flows, infrastructure and network relationships of each TopCare hospital influenced collaboration with academia [cf. 37, 58. Increased coherence between the different forms of boundary work led to productive research collaborations and generated research impact. To meet future requirements, such as regionalization, further boundary work is needed to create long-term collaborations and new ways of rewarding research collaboration within organizations.

## Data Availability

The datasets used and/or analysed during the study are available from the corresponding author upon reasonable request.

## Appendix 1

See Fig. 4 and Tables 5, 6, 7, 8, 9, 10 and 11.

**Table 5:** Overview of bibliometric indicators per hospital (and hospital unit) during the time periods considered

Indicator	Dimension	Definition
*P*	Output	Number of papers (normal articles and reviews) published in journals processed for the WoS
MCS	Impact	Average number of citations per publication, excluding self-citations
MNCS^a,b,c^	Impact	Mean normalized citation score (in comparison with other hospitals)
MNJS	Journal impact	Mean normalized journal score
PP (top 10%)	Impact	Percentage of papers that are among the 10% most frequently cited of all similar papers during the time period considered
PP (uncited)	Overall	Percentage of articles not cited during the time period considered, excluding self-citations
PP (self-citations)	Overall	Percentage of self-citations; a self-citation is defined as a citation in which the citing and cited paper have at least one author in common (first author or co-author)

^a^If the MNCS is higher (or lower) than 1, then on average, the output of the domain is cited more often (or less often) than an “average” publication in the research area in which the domain is active. As with the MNCS, the MNJS indicates the authors’ choice of journal: when the MNJS is far above 1, the author publishes in journals with a high impact in their research specialism, while a score below 1 indicates that the journal has a somewhat lower standing in that specialism

^b^The impact indicators MNCS, MNJS and PP (top 10%) are based on a new method of normalization. While previously normalization was based on WoS Journal Subject categories, we have now moved towards a method in which science is grouped into roughly 4000 clusters, allowing for a like-with-like comparison in which the impact is compared on a much lower scale than the WoS JSCs (Journal Subject Categories), which often contain sub-specialisms with rather divergent citation cultures, leading to inaccurate densities in citation traffic. This created an unfair situation, which the current methodology resolves [62]

^c^In the bibliometric analysis applied for all 8 UMCs and 28 non-academic hospitals, we have added one additional year to the overall citation window for the set of publications. At the time of the study, we selected the papers published in the 2009–2018 period. While 2019 was available at that moment, including 2019 publications would mean providing only 1 year of the citation window to the set of 2019 publications. This means that the citation numbers would be biased within that year towards the ones published earliest in the year, with the first-quarter publications having a clear citation advantage over the publications published later in the year. This would have a disproportional influence on the overall citation analysis, and that is why we decided in the CWTS methodology to always exclude the papers from the final year of a time period, but include that year for citation analysis of the previous years

**Table 6:** Details of document analysis

Overview of documents analysed
- Letters to Parliament and documents from the Ministry of Health
- ZonMw’s website, letters and reports related to the TopCare program
- Objectives and results of the domains and the projects, such as proposals and reports from the two TopCare non-academic hospitals
- Developments in the field, including documents from the Association of Top Clinical Teaching Hospitals (STZ), the Netherlands Federation of University Medical Centers (NFU) and advisory bodies
- Media reports related to the TopCare program
- International and Dutch journals referencing the TopCare program
- Publications evaluating the Collaborations for Leadership in Applied Health Research and Care (CLAHRC)

**Table 7:** Themes and their related codes (interviews)

Themes	Codes
The relationship between a specific domain and the focus of the TopCare hospital: in a specific domain; scientists are committed to the quality of life of patients, which attracts more of these patients to the hospital and influences its focus. This relationship can impact the strategic value of the hospital.	Organizational boundary work: identity [a] Enhancing hospitals’ value proposition
High volume of patients in TopCare hospitals to create a new or recognizable and valuable position.	Organizational boundary work: identity (a) Enhancing hospitals’ value proposition
Using their expertise within a domain to create a new position or enhance their current position.	Organizational boundary work: identity (a) Enhancing hospitals’ value proposition
Unique history of a domain to create a new position or enhance their current position (negative impact: past conflicts may still influence collaboration in the present; positive impact: having a long history within a domain).	Organizational boundary work: identity (a) Enhancing hospitals’ value proposition
Using program funding to enhance the hospitals’ research infrastructure.	Organizational boundary work: competence (a) Enhancing research infrastructures
Finding alignments across domains within hospital and research networks in order to share and learn.	Organizational boundary work: competence (b) Finding alignments within hospitals and research networks
Expanding national and international research networks to share and learn within a domain.	Organizational boundary work: competence (b) Finding alignments within hospitals and research networks
TopCare hospital became interlocutor to enhance or find and mobilize a new strategic academic partner.	Organizational boundary work: power (a) Enhancing the relationship with or finding and mobilizing strategic academic partners
Board of directors and administrators of the two TopCare hospitals work together to learn from each other	Organizational boundary work: power (b) Aligning with the board of directors and administrators of the TopCare hospitals
The person who is ultimately responsible within a hospital’s strategic domain focus for specialized treatments that can only be carried out by a highly qualified team of experts and in specialized facilities.	Boundary spanner: ultimately responsible for a highly qualified team of experts and specialized facilities within the hospital’s strategic domain focus Organizational boundary work: identity (a) Enhancing hospitals’ value proposition
The person who is a leading scientist in a non-academic hospital and the driving force in the collaboration.	Boundary spanner: leading scientist Organizational boundary work: competence (b) Finding alignments within hospitals and research networks
The key figure is often a professor who has a double affiliation (in a non-academic hospital and a UMC) and orchestrates the engagements within these hospitals.	Boundary spanner: double affiliation Organizational boundary work: power (a) Enhancing the relationship with or finding and mobilizing strategic academic partners

**Table 8:** Types of collaboration on publications between UMCs and non-academic hospitals in the Netherlands

	2009–2018/2019					
Types of collaboration on publications	Total number of publications					Joint publication of UMC and non-academic hospital
	UMCs (*n* = 8)	Percentage (%)	Non-academic hospitals (*n* = 28)	Percentage (%)	Total	
Single institution (SI)	27,592	18	1503	8	29,095 (20%)	
National collaboration (NC)	42,557	28	10,880	60	53,436 (31%)	8943
International collaboration (IC)	82,540	54	5896	32	88,435 (52%)	3874
Total	152,688	100	18,279	100	170,967 (100%)	12,816

- UMCs produce 18 times (= 27,592/1503) more SI, four times (= 42,557/10880) more NC and 14 times (82,540/5896) more IC publications than non-academic hospitals.
- Of all publications, 89% (= 152,688/170967) are attributed to UMCs and 11% (18,279/170967) to non-academic hospitals.
- Joint publications in national collaboration: 82% (= 8943/10880) non-academic hospitals and 21% (= 8943/42557) UMCs.
- Joint international publications: 66% (= 3874/5896) non-academic hospitals and 5% (= 3874/82540) UMCs.
- Joint publications: 70% (= 12,816/18279) non-academic hospitals and 8% (= 12,816/152688) UMCs.
- Relationship between joint publications and total publications in each type of collaboration: 17% (= 8943/53436) national collaboration and 4% (= 3874/88435) international collaboration.

**Table 9:** Number of publications in the two periods (%)

	2009–2012/2013				2014–2017/2018			
	UMCs	Percentage (%)	Non-academic hospitals	Percentage (%)	UMCs	Percentage (%)	Non-academic hospitals	Percentage (%)
Single institution	11,204	22	704	11	11,085	16	559	7
National collaboration	15,468	30	4060	63	18,087	26	4556	57
International collaboration	24,133	48	1656	26	39,493	58	2908	36
Total	50,805	100	6420	100	68,665	100	8022	100

The numbers in this table cannot be compared with the totals in Table 8, because the publication year 2013 is missing in the trend analysis

**Table 10:** MNJS

	2009–2018/2019					2009–2012/2013				2014–2017/2018			
	UMCs		Number	Non-academic hospitals	Number	UMCs	Number	Non-academic hospitals	Number	UMCs	Number	Non-academic hospitals	**Number**
SI	1.23		27,592	0.91	1503	1.29	11,204	0.95	704	1.22	11,085	0.90	559
NC	1.24		42,557	1.25	10,880	1.30	15,468	1.33	4060	1.21	18,087	1.21	4556
IC	1.78		82,450	1.93	5896	1.82	24,133	1.95	1656	1.76	39,493	1.96	2908

The MNJS indicates the authors’ choice of journal: when the MNJS is far above 1, the author publishes in journals with a high impact in their research specialism, while a score below 1 indicates that the journal has a somewhat lower standing in that specialism. “Number” indicates total number of publications. SI, Single institution; NC, National collaboration; IC, International collaboration

**Table 11:** MNCS of the four domains in the TopCare program during the period 2010–2016

	MNCS	Total number of publications
Non-academic hospital #1		
Single institution	0.94	72
National collaboration	1.37	196
International collaboration	3.44	164
Non-academic hospital #2		
Single institution	1.68	29
National collaboration	1.64	106
International collaboration	5.85	42

## Abbreviations

BoD: Board of directors
CWTS: Center for Science and Technology Studies
IC: International collaboration
MNCS: Mean normalized citation score
MNJS: Mean normalized journal score
NC: National collaboration
NFU: Netherlands Federation of University Medical Centers
SI: Single institution
STZ: Association of Top Clinical Teaching Hospitals
UMC: University medical centre

## Footnotes

1 The names of the UMCs and non-academic hospitals and their numbers are not up to date due to mergers in the intervening period. The database contains data on eight UMCs; today there are seven, as two UMCs in Amsterdam merged in 2018. There are 28 non-academic hospitals in the database, whereas today 27 such hospitals are members of the Association of Top Clinical Teaching Hospitals (https://www.stz.nl). To ensure data consistency, the database remains unchanged.
